# Protective Effects of Short-Chain Fatty Acids on Endothelial Dysfunction Induced by Angiotensin II

**DOI:** 10.3389/fphys.2020.00277

**Published:** 2020-04-16

**Authors:** Iñaki Robles-Vera, Marta Toral, Néstor de la Visitación, Nazaret Aguilera-Sánchez, Juan Miguel Redondo, Juan Duarte

**Affiliations:** ^1^Department of Pharmacology, School of Pharmacy and Center for Biomedical Research (CIBM), University of Granada, Granada, Spain; ^2^Gene Regulation in Cardiovascular Remodeling and Inflammation Group, Centro Nacional de Investigaciones Cardiovasculares (CNIC), Madrid, Spain; ^3^CIBERCV, Madrid, Spain; ^4^Instituto de Investigación Biosanitaria de Granada, Granada, Spain

**Keywords:** endothelial dysfunction, short-chain fatty acids, nitric oxide, angiotensin II, oxidative stress

## Abstract

Short-chain fatty acids (SCFAs) are among the main classes of bacterial metabolic products and are mainly synthesized in the colon through bacterial fermentation. Short-chain fatty acids, such as acetate, butyrate, and propionate, reduce endothelial activation induced by proinflammatory mediators, at least in part, by activation of G protein–coupled receptors (GPRs): GPR41 and GPR43. The objective of the study was to analyze the possible protective effects of SCFAs on endothelial dysfunction induced by angiotensin II (AngII). Rat aortic endothelial cells (RAECs) and rat aortas were incubated with AngII (1 μM) for 6 h in the presence or absence of SCFAs (5–10 mM). In RAECs, we found that AngII reduces the production of nitric oxide (NO) stimulated by calcium ionophore A23187; increases the production of reactive oxygen species (ROS), both from the nicotinamide adenine dinucleotide phosphate oxidase system and the mitochondria; diminishes vasodilator-stimulated phosphoprotein (VASP) phosphorylation at Ser^239^; reduces GPR41 and GPR43 mRNA level; and reduces the endothelium-dependent relaxant response to acetylcholine in aorta. Coincubation with butyrate and acetate, but not with propionate, increases both NO production and pSer^239^-VASP, reduces the concentration of intracellular ROS, and improves relaxation to acetylcholine. The beneficial effects of butyrate were inhibited by the GPR41 receptor antagonist, β-hydroxybutyrate, and by the GPR43 receptor antagonist, GLPG0794. Butyrate inhibited the down-regulation of GPR41 and GPR43 induced by AngII, being without effect acetate and propionate. Neither β-hydroxybutyrate nor GLPG0794 affects the protective effect of acetate in endothelial dysfunction. In conclusion, acetate and butyrate improve endothelial dysfunction induced by AngII by increasing the bioavailability of NO. The effect of butyrate seems to be related to GPR41/43 activation, whereas acetate effects were independent of GPR41/43.

## Introduction

Vascular endothelial cells are critically involved in cardiovascular homeostasis by controlling thrombotic, inflammatory, and atherogenic states within vascular wall (Thomas et al., 2008). Endothelial dysfunction is typified by a diminution of availability of nitric oxide (NO), accompanied by high levels of reactive oxygen species (ROS), a stimulation in the secretion of proinflammatory cytokines along with a rise in the expression of adhesion molecules (Cai and Harrison, 2000; Hadi et al., 2005; Touyz and Briones, 2011). Endothelial dysfunction, defined as a loss of endothelium to induce vasodilatation, is an earliest indicator of development of cardiovascular disease and appears in the onset and during the progression of hypertension, atherosclerosis, cardiac ischemia, or stroke (Versari et al., 2009; Dharmashankar and Widlansky, 2010; Gkaliagkousi et al., 2015; Vasquez et al., 2019).

Angiotensin II (AngII) is the major effector peptide of renin–angiotensin system. This peptide is a strong stimulus for nicotinamide adenine dinucleotide phosphate (NADPH) oxidase, the main source of ROS in the vascular wall, which induces vascular oxidative stress and endothelial dysfunction (Virdis et al., 2011). In addition, chronic exposure to AngII induces hypertension and vascular remodeling associated to an increased production of NADPH oxidase-derived ROS (Cifuentes et al., 2000).

The gut microbiota is considered important for cardiovascular health (Tang et al., 2019). An ever-growing body of evidence has implicated intestine dysbiosis in the development of hypertension (Yang et al., 2015; Adnan et al., 2017; Li et al., 2017; Toral et al., 2019a, b). Bacterial metabolites mediate interactions with the host. Through resorption and distribution, they can affect intestinal health, and distant immune system functions, the vasculature, and the heart, as well (Bartolomaeus et al., 2019). Short-chain fatty acids (SCFAs) are one of the principal classes of bacterial metabolites. Under physiological conditions, SCFA levels in peripheral blood are very low (in the μM to low mM range) due to hepatic metabolism, with acetate being the main SCFA in circulation (Peters et al., 1992). Nevertheless, high-fiber-diet consumption increased total SCFA levels in serum, with acetate being the most abundant (300 μM) (Thorburn et al., 2015). Moreover, administration of the prodrug of butyrate (Edelman et al., 2003), or intraperitoneal injection of propionate (1 g/kg) (Kimura et al., 2011), or SCFA intravenously (McAndrew et al., 1999), or parenteral nutrition supplemented with 9 mM butyrate (Jirsova et al., 2019) raises SCFA concentrations to high μM or mM levels in blood, which could present a clinical application. Interestingly, chronic AngII infusion to rat induced hypertension accompanied by a decrease in acetate- and butyrate-producing bacteria (Yang et al., 2015), and plasma butyrate was relatively depleted in hypertensive patients (Kim et al., 2018). Butyrate, acetate, and propionate have properties of an antihypertensive character (Natarajan and Pluznick, 2014; Pluznick, 2014; Natarajan et al., 2016). These metabolic products modulate blood pressure (BP) via the vascular and renal G-protein–coupled receptor 41 (GPR-41), and GPR-43 (which lower BP upon stimulation) and olfactory receptor 59 (capable of elevating BP) (Pluznick, 2013). For instance, chronic butyrate treatment improved acetylcholine-induced vasorelaxation and attenuated hypertension induced by AngII infusion in mice (Kim et al., 2018). Acetate supplementation was able to prevent the development of hypertension and heart failure in a deoxycorticosterone acetate–salt murine model of hypertension (Marques et al., 2017). The SCFA propionate protects from AngII-induced cardiac and vascular damage in AngII-infused apolipoprotein E knockout (ApoE^–/–^) mice (Bartolomaeus et al., 2019). On the other hand, oral supplementation with butyrate is able to decrease oxidative stress in atherosclerotic lesion sites, attenuating endothelial dysfunction and macrophage migration and activation in ApoE^–/–^ mice (Aguilar et al., 2016). Furthermore, SCFAs play a beneficial role reducing endothelial activation, which leads to a reduction in cytokine production and adhesion molecules expression (Li et al., 2018a, b). Short-chain fatty acids might modulate endothelial function either by activating GPRs (Vinolo et al., 2011) and/or inhibiting histone deacetylases (HDACs). However, whether the protective effects in vascular oxidative stress and endothelial dysfunction induced by chronic SCFAs in AngII-infused animals are related to direct effects in the endothelium is unknown. Thus, the objective of the present study was to examine the direct effects of SCFAs against endothelial dysfunction induced by AngII in both rat aortic endothelial cells (RAECs) and intact rat thoracic aorta and the role of GPR41/43.

## Materials and Methods

### Vascular Reactivity Studies

Descending thoracic aortic segments from rats were maintained for 24 h in Medium 199 (Gibco, Invitrogen Life Technologies, Carlsbad, CA, United States) + 20% fetal bovine serum + amphotericin B 2 mM + penicillin/streptomycin 2 mM + glutamine 2 mM + HEPES 10 mM, all from Sigma-Aldrich, Barcelona, Spain) at 37°C under 5% CO_2_ (Robles-Vera et al., 2018). During this time, the rings were incubated with acetate (10 mM), propionate (10 mM), or butyrate (5 mM), and in the last 6 h in AngII-free condition (control rings) or under AngII (1 μM)–stimulated condition serum-free Medium 199. In some experiments, rings were coincubated for 24 h with the antagonist of GPR-43 receptor, GLPG0974 (GLPG) (Namour et al., 2016) (Bio-Techne R&D Systems, S.LU, Madrid, Spain) (0.1 μM), or with β-hydroxybutyrate (SHB), a ketone body and antagonist of GPR-41 (5 mM) (Kimura et al., 2011) (Sigma-Aldrich). After the incubation period, rings were mounted in organ chambers filled with Krebs solution (2 mM CaCl_2_, 1.2 mM KH_2_PO_4_, 118 mM NaCl, 25 mM NaHCO_3_, 4.75 mM KCl, 1.2 mM MgSO_4_ and 11 mM glucose) at 37°C and gassed with 95% O_2_ and 5% CO_2_ (Sanchez et al., 2007). The concentration–relaxation response curves to acetylcholine (ACh, 1 nM to 10 μM) were performed in rings precontracted by 1 μM phenylephrine (Phe). Relaxant responses to ACh were expressed as a percentage of the response to Phe.

### Primary Culture of Rat Aortic Endothelial Cells

Rat aortic endothelial cells were obtained from rat thoracic aortas as previously reported (Toral et al., 2015) with some modifications. The resulting cells were cultured [Medium 199 (Gibco, Invitrogen Life Technologies) + 20% fetal bovine serum + amphotericin B 2 mM + penicillin/streptomycin 2 mM + glutamine 2 mM + HEPES 10 mM + heparin 100 mg/mL + endothelial cell growth supplement 30 μg/mL; all from Sigma-Aldrich] at 37°C and 5% CO_2_. Rat aortic endothelial cells were incubated with acetate (10 μM to 10 mM), propionate (10 mM), or butyrate (1 μM to 5 mM), in serum free Medium 199 for 24 h. For the last 6 h in the appropriated wells, AngII (1 μM) was added to induce a stimulated condition. In some experiments, cells were coincubated for 24 h with the GPR-43 antagonist GLPG (0.1 μM), or with the GPR-41 antagonist, SHB (5 mM). After the incubations, RAECs were used to analyze NO, ROS production, mitochondrial ROS production, NADPH oxidase activity, and gene and protein expression levels.

### Quantification of NO Released by Diaminofluorescein 2

A NO-sensitive fluorescent probe, diaminofluorescein 2 (DAF-2) (Sigma-Aldrich), was employed for the quantification of NO released by RAECs as previously described (Toral et al., 2015). Succinctly, RAECs were incubated in 96-well plates, as already mentioned. Then, cells were washed with phosphate-buffered saline (PBS) + 0.9 mM Ca^2+^ and then were preincubated with L-arginine (100 μM in PBS, 5 min, 37°C) (Sigma-Aldrich). Afterward, DAF-2 (0.1 μM) was added for 2 min, and then a calcium ionophore, calimycin (A23187, 1 μM) (Sigma-Aldrich), was incubated for 30 min. Subsequently, fluorescence intensity [expressed as arbitrary units (AU)] was determined at excitation and emission wavelengths of 490 and 510 nm, respectively, using a spectrofluorometer (Fluorostart; BMG Labtechnologies, Offenburg, Germany). Autofluorescence was subtracted from all values. To determine NO-independent fluorescence signal induced by A23187, N^G^-nitro-L-arginine methyl ester (L-NAME, 100 μM) was added to some wells 15 min before the addition of L-arginine. The difference between the fluorescence signal with and without L-NAME was considered as NO production.

### Immunofluorescence in Cultured Cells

Rat aortic endothelial cells were then plated on gelatin-coated plates and were incubated under the already mentioned conditions. Cells were stimulated by the calcium ionophore calimycin (A23187, 1 μM) for 30 min. Then, cells were fixed in 4% formaldehyde in PBS for 10 min and permeabilized with 0.5% Triton X-100 in PBS for 15 min. Cells were then stained with antibody to mouse monoclonal anti–p-VASP-Ser^239^; 1:50; Santa Cruz Biotechnology, Santa Cruz, CA, United States). Specificity was determined by substituting primary antibody with unrelated immunoglobulin G (Santa Cruz Biotechnology). The secondary antibody was Alexa-Fluor-546–conjugated goat anti–mouse (BD Pharmingen, Franklin Lakes, NJ, United States). Sections were mounted with DAPI in Citifluor AF4 mounting medium (Aname, Madrid, Spain), as previously described (Du et al., 2012). Images were acquired at 1,024 × 1,024 pixels, 8 bits, using a Leica SP5 (Madrid, Spain) confocal microscope with 40 × oil-immersion objective. The laser intensity and exposure time were kept identical for all measurements.

### Measurement of Intracellular ROS Concentrations

The fluorescent probe 5-(and-6-)chloromethyl-2′-7′-dichlorodihydrofluorescein diacetate (CM-H2DCFDA) (Invitrogen Life Technologies) was used to measure endothelial ROS production. Confluent RAECs in 96-well plates were incubated as described before. To assess the role of NADPH oxidase in ROS production, some cells were also coincubated with the selective NADPH oxidase inhibitor VAS2870 (10 μM) (Sigma-Aldrich). During this period, the cells were incubated with 5 μM CM-H2DCFDA for 30 min at 37°C. After the incubation, the cells were washed twice with PBS + 0.9 mM Ca^2+^. Fluorescence levels were determined at excitation and emission wavelengths of 490 and 545 nm, respectively, with a spectrofluorometer (Fluorostart; BMG Labtechnologies) (Toral et al., 2015).

### Mitochondrial ROS Measurement

Mitochondrial ROS production was determined through an already described method adequately modified for these specifications (Wojtala et al., 2014), using the MitoSOX^TM^ Red (Invitrogen Life Technologies), a mitochondrial O_2_^–^ molecular sensor. Confluent RAECs were incubated as reported before. Afterward, MitoSOX^TM^ Red (5 μM) was added; some cells were also coincubated with MitoQ (0.1 μM) (donated by Dr. M. P. Murphy, Medical Research Council Mitochondrial Biology Unit, Cambridge, United Kingdom) (selective scavenger of mitochondrial ROS), and cells were incubated in the dark, 30 min at 37°C. After, the media was removed, and the washing of the cells with PBS + 0.9 mM Ca^2+^, fluorescent intensity (expressed as relative fluorescent units), was determined through excitation and emission wavelengths of 490 and 590 nm, respectively, employing a spectrofluorometer (Fluorostart; BMG Labtechnologies).

### NADPH Oxidase Activity

The NADPH oxidase activity in intact RAECs was also determined, as previously described, by dihydroethidium (DHE) fluorescence assay in the microplate reader (Toral et al., 2015). Confluent RAECs grown in 6-well dishes (well area of 9.6 cm^2^) incubated as previously described were washed with cold PBS; harvested; homogenized using lysis buffer composed of 50 mM Tris, pH 7.4, containing 0.1 mM EDTA, 0.1 mM EGTA, 10 μg/mL aprotinin, 10 μg/mL leupeptin, and 1 mM phenylmethysulfonyl fluoride; and sonicated (10 s of 3 cycles al 8W). Ten micrograms of protein obtained from fresh homogenates was incubated with DHE (10 μM) and DNA (1.25 μg/mL) in phosphate buffer (100 mM), pH 7.4, containing 100 μM diethylenetriamine pentaacetic acid (PBS/DTPA) with the addition of NADPH (50 μM), at a final volume of 120 μL. Incubations were performed for 30 min at 37°C, in the presence or absence of VAS2870 (10 μM), in the absence of light. Total fluorescence was measured in a microplate reader using a rhodamine filter (excitation 490 nm and emission 590 nm) with a spectrofluorometer (Fluorostart; BMG Labtechnologies).

### Reverse Transcriptase–Polymerase Chain Reaction and Western Blot Analysis

Reverse transcriptase–polymerase chain reaction (RT-PCR) analysis was performed using standard methods; total RNA was extracted from RAECs by homogenization and converted to cDNA. Cells were scratched and harvested in 0.5 mL of Tri Reagent (Thermo Fisher Scientific, Waltham, MA, United States). RNA isolation was performed through conventional methods consisting of sequential washes using bromochloropropane, isopropanol, and ethanol 75%. RNA concentrations were determined with a NanoDrop 2000 Spectrophotometer (Thermo Fisher Scientific). Polymerase chain reaction was carried out with a Techne Techgene thermocycler (Techne, Cambridge, United Kingdom). Quantitative real-time RT-PCR was applied to analyze mRNA expression. The utilized forward and reverse primer sequences are included in Table 1. Previous experiments were conducted with different amounts of cDNA in order to find non-saturating conditions of PCR amplification for all genes studied. The relative quantification of mRNA was judged by the SYBR Green–based RT-PCR method. The quality of the PCR reaction was determined according with dilution series of a standard cells sample. The ΔΔCt method was used for quantification. For internal normalization, the housekeeping gene GAPDH was selected.

**TABLE 1:** Oligonucleotides for real-time RT-PCR.

mRNA targets	Descriptions	Sense	Antisense
NOX-1	NOX-1 subunit of NADPH oxidase	TCTTGCTGGTTGACACTTGC	TATGGGAGTGGGAATCTTGG
*p47phox*	p47phox subunit of NADPH oxidase	CCCAGCGACAGATTAGAAGC	TGGATTGTCCTTTGAGTCAGG
*GPR-41*	G-protein-coupled receptor-41	TGACGGTGAGCATAGAACGTTT	GCCGGGTTTTGTACCACAGT
*GPR-43*	G-protein-coupled receptor-43	TCGTGGAAGCTGCATCCA	GCGCGCACACGATCTTT
*GAPDH*	Glyceraldehyde-3-phosphate dehydrogenase	ACCACAGTCCATGCCATCAC	TCCACCACCCTGTTGCTGTA

Western blotting was performed as described previously (Toral et al., 2015). Rat aortic endothelial cell homogenates were run on using sodium dodecyl sulfate–polyacrylamide gel electrophoresis. Proteins were transferred to polyvinylidene difluoride membranes and incubated with primary antibody to mouse monoclonal anti-p-VASP-Ser^239^, rabbit monoclonal anti-peNOS-ser-1177 antibody (Cell Signaling Technology, Danvers, MA, United States), or mouse monoclonal anti-eNOS (Endothelial nitric oxide synthase) antibody (Transduction Laboratories, San Diego, CA, United States), and mouse monoclonal anti–α-actin (Sigma-Aldrich) antibodies overnight and with the corresponding secondary peroxidase-conjugated antibodies. Antibody binding was detected by an ECL system (Amersham Pharmacia Biotech, Amersham, United Kingdom), and densitometric analysis was performed using Scion Image-Release Beta 4.02 software^1^.

### Statistical Analysis

All data are presented as mean ± SEM. Statistical analyses were carried out with the GraphPad Prism 7 software (GraphPad Software, San Diego, CA, United States). The Shapiro–Wilk test was performed for normally distributed continuous variables. Statistical comparisons were done using the one-way analysis of variance with Dunnett procedure for *post hoc* analysis for parametric analysis or the Kruskal–Wallis test for non-parametric analysis. Significance was considered for values of *p* < 0.05.

## Results

### Butyrate and Acetate Improve AngII-Induced Impairment of Endothelium-Dependent Vasodilation and NO Production

**FIGURE 1:**
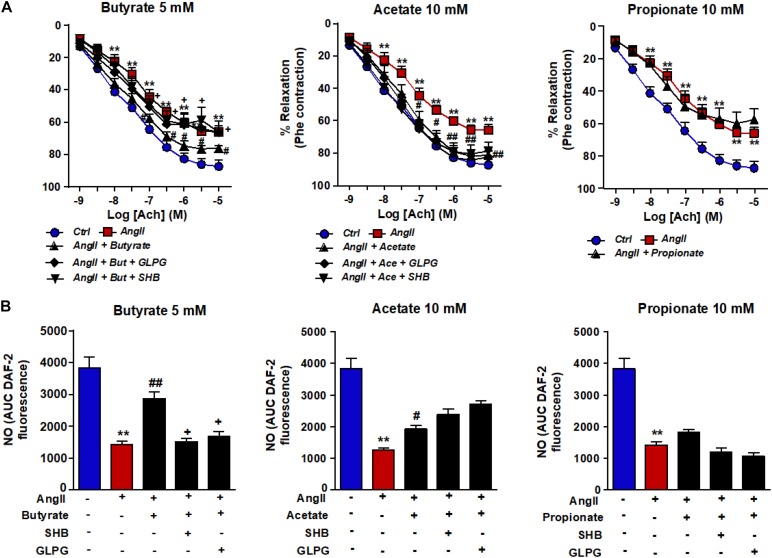
Effects of short-chain fatty acids (SFCAs) in angiotensin (Ang) II–induced endothelial dysfunction *in vitro*. Vascular relaxant responses induced by acetylcholine (ACh) **(A)** in rat aortas precontracted by phenylephrine (Phe, 1 μM) incubated with butyrate (5 mM), acetate (10 mM), or propionate (10 mM). In some experiments, aortic rings were coincubated with GLPG0974 (GLPG, 0.1 μM) or β-hydroxybutyrate (SHB, 5 mM) for 24 h, and in the last 6 h in the absence (Ctrl) or presence of AngII (1 μM). Results are shown as mean ± SEM, derived from six to eight different rings. *P < 0.05 and **P < 0.01 vs. ctrl; ^#^*P* < 0.05 and ^##^*P* < 0.01 vs. AngII; ^+^*P* < 0.05 vs. AngII + SFCA group. Nitric oxide (NO) release stimulated by A23187 **(B)** in RAECs incubated with butyrate (5 mM), acetate (10 mM), or propionate (10 mM). In some experiments, cells were coincubated with GLPG0974 (GLPG, 0.1 μM) or SHB (5 mM) for 24 h, and in the last 6 h in the absence (control) or presence of angiotensin (Ang)II (1 μM). Nitric oxide release was estimated from the area under the curve (AUC) of the fluorescent signal of 4,5-diaminofluorescein (DAF-2) for 30 min of stimulation. Results are shown as mean ± SEM (six to eight). **P < 0.01 vs. ctrl; ^#^*P* < 0.05 and ^##^*P* < 0.01 vs. AngII; ^+^*P* < 0.05 vs. AngII + SFCA group.

**FIGURE 2:**
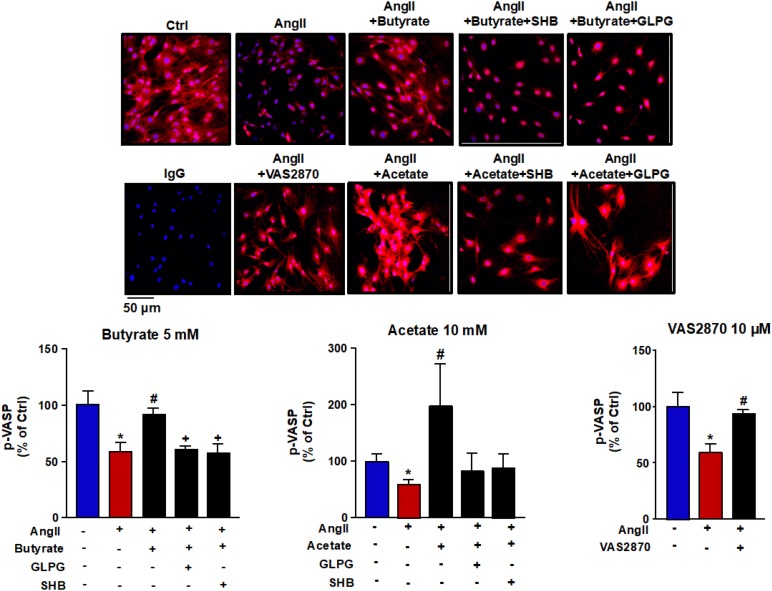
Effects of short-chain fatty acids (SFCAs) in VASP phosphorylation in rat aortic endothelial cells (RAECs). Pictures show VASP (Ser^239^) phosphorylation in RAECs incubated with butyrate (5 mM), acetate (10 mM), or propionate (10 mM). In some experiments, cells were coincubated with GLPG0974 (GLPG, 0.1 μM) or β-hydroxybutyrate (SHB, 5 mM) for 24 h, and in the last 6 h in the absence (Ctrl) or presence of angiotensin (Ang)II (1 μM), measured by immunostaining (red fluorescence) intensity normalized to the blue nuclear stain DAPI. Cells were stimulated by the calcium ionophore calimycin (A23187, 1 μM) for 30 min. Scale bar, 50 μm. Results are shown as mean ± SEM (four to six). *P < 0.05 vs. ctrl; ^#^*P* < 0.05 vs. AngII; ^+^*P* < 0.05 vs. AngII + SFCA group.

Incubation of rat aortas with AngII inhibited the endothelium-dependent relaxation to ACh(Figure 1A). In RAECs, AngII reduced the A23187-stimulated NO production (Figure 1B) and VASP phosphorylation at Ser^239^ (Figure 2). Supraphysiological concentrations of butyrate and acetate treatment, but not propionate, improved the aortic relaxation to ACh (Figure 1A), A23187-stimulated NO generation (Figure 1B), and VASP-Ser^239^ phosphorylation (Figure 2) in aorta and RAECs exposed to AngII, respectively. We also analyzed the effects of physiological plasma concentrations of butyrate and acetate in NO generation impaired by AngII in RAECs. We found that concentrations of butyrate ≥100 μM and acetate ≥1 mM were able to prevent the reduction in NO production induced by AngII (Supplementary Figure S1). The protective effects of butyrate (5 mM) on VASP-Ser^239^ phosphorylation in RAECs were also confirmed by Western blot (Supplementary Figure S2A). Because GPR-41 and GPR-43 are the two targets of SCFAs (Ang and Ding, 2016), we found that the effect of butyrate was inhibited by coincubation with the antagonist of GPR-41, SHB, and by the antagonist of GPR-43, GLPG (Figures 1, 2). Nonetheless, the effect of acetate on endothelial dysfunction and NO release by endothelial cells was not abrogated by any of the antagonists (Figures 1, 2). When we analyzed eNOS phosphorylation at the active site Ser^1177^, we found that AngII was unable to reduce significantly the ratio peNOS^Ser1177^/eNOS as compared the control, and the presence of butyrate did not modify the level of phosphorylation (Supplementary Figure S2B). In order to examine whether NADPH oxidase–driven ROS production is involved in endothelial dysfunction induced by AngII, we measured the relaxation to ACh in aorta (Supplementary Figure S3) and the phosphorylation of VASP at Ser^239^ by immunofluorescence in RAECs incubated with the specific pan-NOX inhibitor VAS2870. We found that both the impaired ACh relaxation (Supplementary Figure S2) and the reduction in VASP phosphorylation induced by AngII were prevented by NADPH oxidase inhibition (Figure 2).

### Butyrate, Acetate, and Propionate Reduce the ROS Production Induced by AngII

**FIGURE 3:**
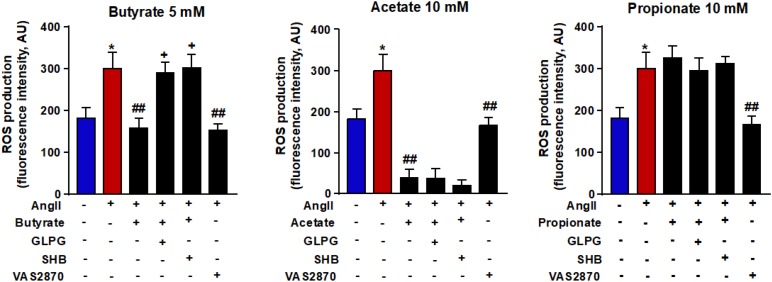
Effects of short-chain fatty acids (SFCAs) in intracellular reactive oxygen species (ROS) production in rat aortic endothelial cells (RAECs). Reactive oxygen species were measured by fluorescence in CM-H2DCFDA-loaded cells in RAECs incubated with butyrate (5 mM), acetate (10 mM), or propionate (10 mM). In some experiments, cells were co-incubated with GLPG0974 (GLPG, 0.1 μM) or β-hydroxybutyrate (SHB, 5 mM) for 24 h, and in the last 6 h in the absence (control) or presence of AngII (100 μM). Results are shown as mean ± SEM (six to eight). **P < 0.01 vs. ctrl; ^##^*P* < 0.01 vs. AngII; ^++^*P* < 0.01 vs. AngII + SFCA group.

**FIGURE 4:**
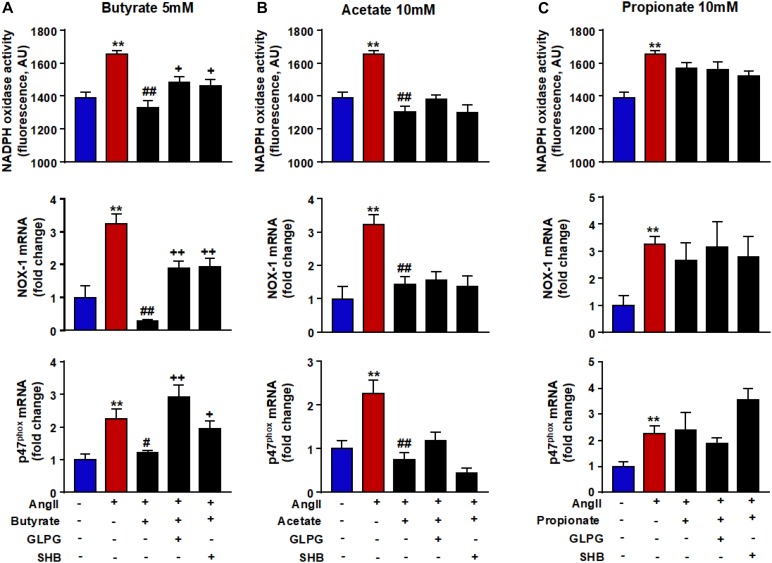
Effects of short-chain fatty acids (SFCAs) in NADPH oxidase in rat aortic endothelial cells (RAECs). NADPH oxidase activity measured by DHE fluorescence and mRNA levels of NADPH oxidase subunits NOX-1 and p47^phox^ in RAECs incubated with butyrate (5 mM, **A**), acetate (10 mM, **B**), or propionate (10 mM, **C**). In some experiments, cells were coincubated with GLPG0974 (GLPG, 0.1 μM) or β-hydroxybutyrate (SHB, 5 mM) for 24 h, and in the last 6 h in the absence (control) or presence of AngII (1 μM). Results are shown as mean ± SEM (six to eight). **P < 0.01 vs. ctrl; ^#^*P* < 0.05 and ^##^*P* < 0.01 vs. AngII; ^+^*P* < 0.05 and ^++^*P* < 0.01 vs. AngII + SFCA group.

Because ROS is closely linked to endothelial dysfunction, we analyzed the intracellular oxidative stress. It was measured using the CM-H2DCFDA fluorescent probe. Compared with control cells, the fluorescence signal in AngII-exposed cells (Figure 3) was significantly ≈1.6-fold higher (181.8 ± 26.2 vs. 299.8 ± 41.4 AU, *P* < 0.05, respectively). This ROS increase was reduced by butyrate (159.0 ± 24.9 AU, *P* < 0.01), acetate (38.9 ± 22.0 AU, *P* < 0.01) but not by propionate (326.1 ± 30.0 AU, *P* > 0.05) (Figure 3). The effect of butyrate was inhibited by coincubation with the antagonist of GPR-41, SHB by ≈100%, and by the antagonist of GPR-43, GLPG by ≈93% (Figure 3). However, the incubation with SHB or GLPG did not change the effects of acetate (19.8 ± 15.2 and 37.9 ± 25.5 AU, respectively, *P* > 0.05 vs. acetate alone) or propionate (311.6 ± 18.8 and 295.8 ± 31.1 AU, respectively, *P* > 0.05 vs. propionate alone) (Figure 3). This ROS increase was inhibited by the VAS2870, showing the critical role of increased NADPH oxidase in the intracellular ROS production stimulated by AngII (Figure 3). Additionally, the NADPH oxidase activity was also increased by AngII, whereas butyrate (Figure 4A) and acetate (Figure 4B) inhibited significantly this increase, with propionate being without effect (Figure 4C). Again both SHB and GLPG inhibited the effects of butyrate, with acetate being without effects (Figure 3). Similarly, butyrate abolished the up-regulation of NOX-1 and p47^phox^ mRNA in RAECs incubated with AngII. Once more, this effect was reversed when combined with SHB or GLPG (Figure 4A). However, the acetate treatment prevented the up-regulation of NOX-1 and p47^phox^ in AngII-treated cells. This effect was not altered by GLPG or SHB (Figure 4B). By contrast, propionate did not affect the significant mRNA increase of the main NADPH oxidase subunits in RAECs incubated with AngII (Figure 4C).

### Butyrate and Acetate Reduce the Mitochondrial ROS Production Induced by AngII

**FIGURE 5:**
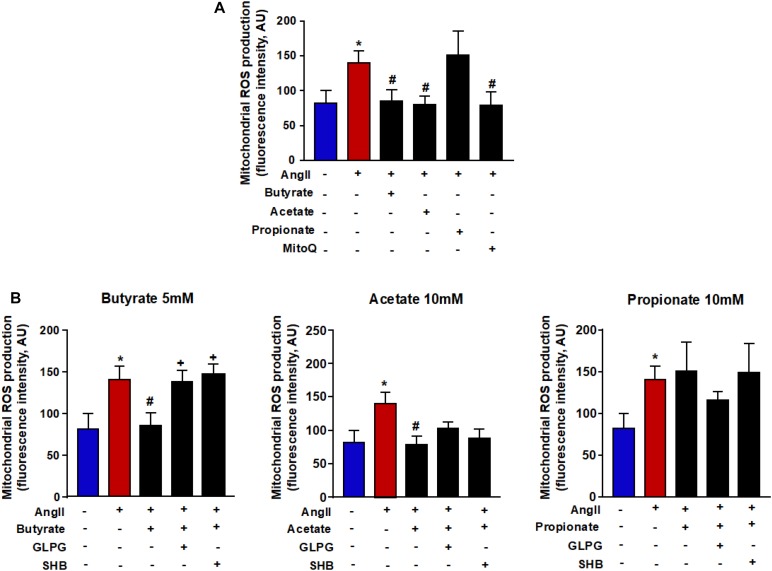
Effects of short-chain fatty acids (SFCAs) in mitochondrial ROS in rat aortic endothelial cells (RAECs). Mitochondrial ROS **(A)** in the presence of the antioxidant mitoQ (0.1 μM) during AngII incubation in RAECs incubated with butyrate (5 mM), acetate (10 mM), or propionate (10 mM). In some experiments **(B)**, cells were coincubated with GLPG0974 (GLPG, 0.1 μM) or β-hydroxybutyrate (SHB, 5 mM) for 24 h, and in the last 6 h in the absence (control) or presence of AngII (1 μM). Results are shown as mean ± SEM (six to eight). *P < 0.05 vs. ctrl; ^#^*P* < 0.05 vs. AngII; ^+^*P* < 0.05 vs. AngII + SFCA group.

Mitochondria are large generators of ROS during the process of oxidative phosphorylation. These ROS are able to bind to NO and reduce its bioavailability. It led us to analyze the action of SCFAs on the mitochondrial ROS production. Mitochondrial ROS were measured by MitoSOX^TM^ Red in RAECs. Its signal was increased after incubation with AngII. This ROS increase was reduced by butyrate and acetate but not by propionate (Figure 5A). To confirm the role of mitochondrial ROS, cells were incubated with the mitochondrial antioxidant mitoQ, which lowered the high ROS production induced by AngII (Figure 5A). The effect of butyrate was abolished by coincubation with the antagonists SHB and GLPG (Figure 5B). However, the effect of acetate was not modulated by any of the antagonists used (Figure 5B).

### Butyrate Prevents GPR-41 and GRP-43 Down-Regulation Induced by AngII in RAECs

**FIGURE 6:**
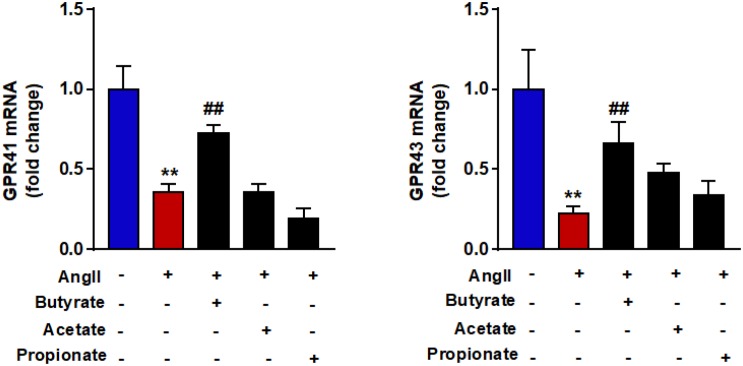
Effects of short-chain fatty acids (SFCAs) in GRPs expression in rat aortic endothelial cells (RAECs). mRNA levels of GRP41 and GRP43 in RAECs incubated with butyrate (5 mM), acetate (10 mM), or propionate (10 mM) for 24 h, and in the last 6 h in the absence (control) or presence of AngII (1 μM). Results are shown as mean ± SEM (six to eight). **P < 0.01 vs. control; ^##^*P* < 0.01 vs. AngII.

To examine if shifts in GPRs expression levels were involved in the protective effects of SCFAs in endothelial function, the mRNA levels of GPR-41 and GPR-43 were measured in RAECs. Angiotensin II induced a significant down-regulation of both GPRs (Figure 6). Coincubation with butyrate but not with acetate or propionate increased mRNA levels of both GPR-41 and GPR-43.

## Discussion

Our research demonstrates, for the first time, that butyrate and acetate, at concentrations raised under pharmacological interventions, improve endothelial dysfunction induced by the prohypertensive agent AngII by increasing the bioavailability of NO involving GPR activation. The principal findings of the present study are the following: (1) butyrate and acetate restored endothelium-dependent vasodilatation in aortic rings and increased calcium ionophore–stimulated NO production by endothelial cells incubated with AngII; (2) both SCFAs decreased intracellular ROS production from NADPH oxidase and mitochondria; (3) these SCFAs restored the phosphorylation of VASP at Ser^239^; and (4) GPR-41 and GPR-43 antagonists inhibit the beneficial effects of butyrate in AngII-induced endothelial dysfunction, without affecting acetate effects.

Endothelial dysfunction presents a characteristic shift of activity from the endothelium toward reduced vasodilation. Nitric oxide is the main endothelium-dependent vasodilator in the aorta. In fact, the incubation with the eNOS blocker L-NAME abolished ACh-induced relaxation in rat aorta (Gómez-Guzmán et al., 2015). Nitric oxide–induced relaxation is linked to high levels of cGMP in vascular smooth muscle cells, an interaction known as the NO-cGMP signaling pathway. Disturbance of NO signaling pathways is one of the major determinants for endothelial dysfunction, which is characterized by the reduction of the NO bioavailability with the resulting impairment of the endothelium-dependent vasodilation (Endemann and Schiffrin, 2004). In all cases, p-VASP-Ser^239^ can be used as a sensitive marker of the NO-cGMP pathway (Wang et al., 2019). Recently, it has been shown that, in vascular tissue where endothelial dysfunction is present and NO bioavailability is decreased, VASP phosphorylation at Ser^239^ was strikingly reduced (Oelze et al., 2000; Mülsch et al., 2001). Mollnau et al. (2002) found that AngII infusion caused endothelial dysfunction in vessels linked to a strong decrease in p-VASP-Ser^239^. It is unlikely that the observed reduction in p-VASP-Ser^239^ was caused by a depletion in substrate availability, because total VASP expression was patently similar in vessels from sham-treated and AngII-infused animals. In agreement with this information, our data showed that p-VASP-Ser^239^ was reduced in AngII-treated endothelial cells. This suggests the involvement of an impaired NO-cGMP pathway in endothelial-dependent smooth muscle relaxation reduction in aortic rings incubated with AngII. Interestingly, butyrate and acetate improved the impaired NO-cGMP pathway. This protective effect of both SCFAs was supported by (i) increased NO production and VASP phosphorylation at Ser^239^ in RAECs and (ii) increased endothelium-dependent vasorelaxation to ACh in aortic rings incubated with AngII. The protective effects of butyrate seem to be independent of increased eNOS activation, because butyrate did not change eNOS phosphorylation at the active site Ser1177. However, we did not find beneficial effects of propionate on endothelial dysfunction induced by AngII. This result suggests that the improvement in vascular dysfunction and hypertension in AngII–infused wild-type NMRI mice and AngII-infused ApoE^–/–^ mice induced by propionate (Bartolomaeus et al., 2019) seems to be independent of direct effects in the NO-GMPc pathway in the vascular wall.

An excessive production of ROS is crucially associated with the breakdown of NO linked to endothelial dysfunction in vascular tissues from AngII-infused rats (Rajagopalan et al., 1996; Virdis et al., 2004). It is well established that endothelial dysfunction induced by AngII was also associated with increased production of ROS from both NADPH oxidase (Griendling et al., 1994; Li and Shah, 2003; Sanchez et al., 2007) and mitochondria (Doughan et al., 2008; Dikalov et al., 2014) in vascular cells. In our experiments, the mitochondrial antioxidant mitoQ and the NADPH oxidase inhibitor VAS2870 prevented the AngII-induced increase in mitochondrial and intracellular ROS, respectively. Interestingly, VAS2870 restored ACh-induced relaxation and p-VASP- Ser^239^ level in AngII-treated aorta and RAECs, respectively, confirming the critical involvement of NADPH oxidase in AngII-induced endothelial dysfunction. The elevated NADPH oxidase activity induced by AngII was associated with increased expression of its subunits NOX-1 and p47^phox^. Butyrate and acetate prevented the increased ROS levels. This effect was caused by reducing both mitochondrial and NADPH oxidase-derived ROS production in endothelial cells. The normalization of ROS also seems to contribute to the restoration of endothelial function. Our results suggest that the decrease in ROS levels in the vascular wall and the ensuing prevention of NO inactivation constitute a pivotal mechanism involved in the SCFAs’ protective effects on endothelial function. The present results agree with previous data showing that attenuation of endothelium dysfunction, macrophage migration, and activation in the atherosclerotic lesion in ApoE^–/–^ mice induced butyrate supplementation linked to a decrease in oxidative stress related to a reduced NADPH oxidase expression (Aguilar et al., 2016).

Short-chain fatty acids might modulate endothelial function either through the inhibition of HDACs and/or the activation of GPRs: GPR-41 and GPR-43 (Vinolo et al., 2011). Activation of GPR41/43 mediated, at least in part, the inhibitory effects of SCFAs in cytokine production stimulated by LPS or TNFα in human umbilical vein endothelial cells (Li et al., 2018b). Our studies found a crucial role of GPR-41 and GPR-43 in the protective effects of butyrate on endothelial dysfunction and oxidative stress induced by AngII. Our results demonstrate that pharmacological blockade of GPR-41 by SHB, and GPR-43 by GLPG, inhibited the protective effects of butyrate on NO production, intracellular ROS accumulation, VASP phosphorylation, and endothelium-dependent relaxation to ACh. Nevertheless, we also found that acetate prevented endothelial dysfunction induced by AngII in a GPR-independent manner, because it was unaffected by its antagonists. A reduced GPR-41 expression in aorta has been associated to reduced plasma butyrate levels and high BP in spontaneously hypertensive rats, as compared to Wistar–Kyoto rats (Robles-Vera et al., 2017; Kim et al., 2018). Interestingly, we found that AngII down-regulated both GPR41 and GPR43 in endothelial cells. This effect was significantly inhibited by butyrate, whereas both acetate and propionate were without effect. This enhanced expression of GPR41/43 induced by butyrate in RAECs might account for its protective effects in endothelial function. However, the interaction between activated GPR41/43 and AngII pathways to prevent endothelial dysfunction has not been addressed in the present study.

## Conclusion

**FIGURE 7:**
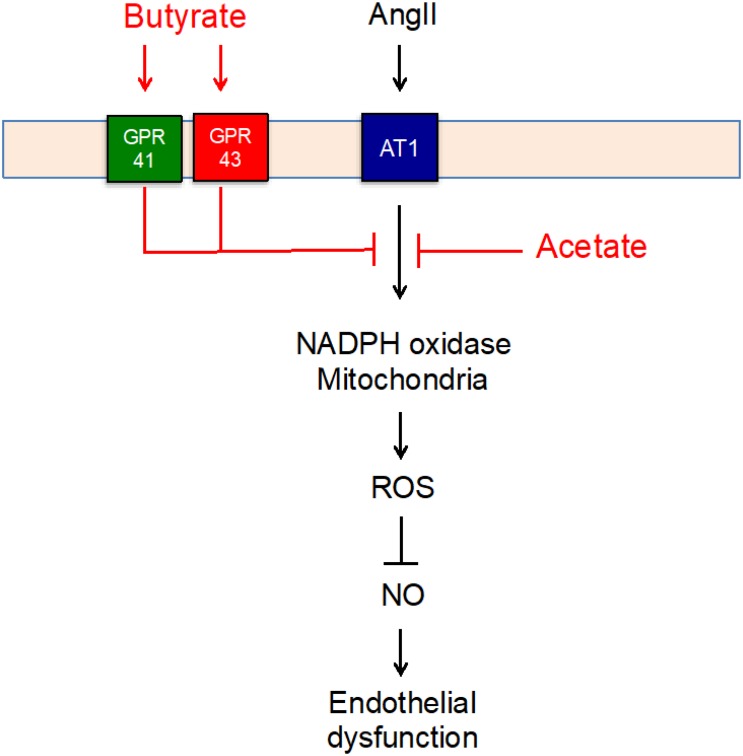
Scheme representing the mechanisms for the protective effect of short-chain fatty acids (SFCAs) (in red) involved in the angiotensin (Ang)II–induced impairment of endothelial function).

In conclusion, butyrate and acetate improved endothelial dysfunction induced by AngII by an increase in NO bioavailability as a result of oxidative stress inhibition. The effects of butyrate seem to be dependent of GPR41/43 activation, whereas the preventive effects of acetate were GPR-independent (Figure 7). These actions in the endothelium might be associated with the vascular protective effect of chronic SCFA supplementation in hypertension and atherosclerosis induced by AngII described previously. In addition, gut microbiota modifications by prebiotics or probiotics addressed to increase plasma acetate and/or butyrate levels might be beneficial to prevent the detrimental effects of AngII in the vascular wall.

## Data Availability Statement

The datasets generated for this study are available on request to the corresponding author.

## Ethics Statement

The experimental protocol was approved by the Ethics Committee of Laboratory Animals of the University of Granada (Spain; permit number 03-CEEA-OH-2013).

## Author Contributions

IR-V, MT, JR, and JD participated in research design. IR-V, MT, NA-S, and NV performed the most of experiments. IR-V, MT, NV, and JD contributed to data analysis. IR-V, MT, JR, and JD wrote or contributed to the writing of the manuscript. All authors approved the final version to be published.

## Conflict of Interest

The authors declare that the research was conducted in the absence of any commercial or financial relationships that could be construed as a potential conflict of interest.

## Footnotes

1 http://www.scioncorp.com

## Abbreviations

A23187: calcium ionophore calimycin
ACh: acetylcholine
AngII: angiotensin II
ApoE^–/–^: apolipoprotein E knockout
AU: arbitrary units
BP: blood pressure
CM-H2DCFDA: 5-(and-6) chloromethyl-2′-7′-dichlorodihydrofluorescein diacetate
DAF-2: diaminofluorescein-2
DHE: dihydroethidium
GLPG: GLPG0974
GPR: G-protein -coupled receptor
HDACs: histone deacetylases
L-NAME: N^G^-nitro-L-arginine methyl ester
NO: nitric oxide
Phe: phenylephrine
RAECs: rat aortic endothelial cells
ROS: reactive oxygen species
SCFAs: short-chain fatty acids
SHB: β-hydroxybutyrate
VASP: vasodilator-stimulated phosphoprotein.
