# Supplementary material for: Protective Effects of Short-Chain Fatty Acids on Endothelial Dysfunction Induced by Angiotensin II
Source: Front Physiol. 2020 Apr 16;11:277. doi: 10.3389/fphys.2020.00277 (PMC7176911; doi:10.3389/fphys.2020.00277)
Supplement: Supplementary file 1 [file Data_Sheet_1.PDF]

Supplementary Material

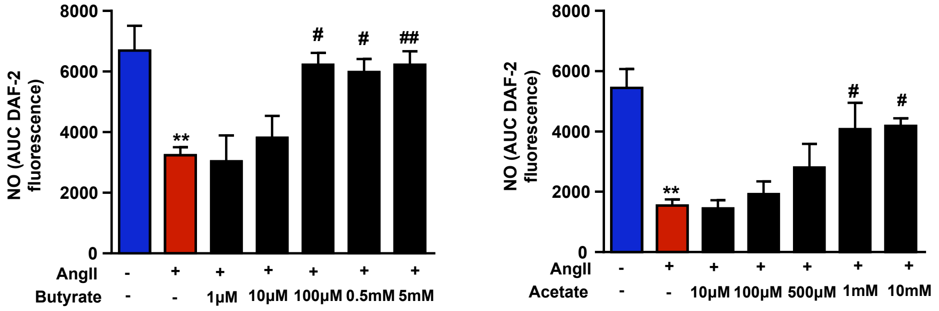

Figure S1. Effects of physiological concentrations of SCFAs in nitric oxide (NO) release stimulated by A23187 in RAECs. Cells were incubated with butyrate or acetate for 24 hours, and in the last 6 hours in the absence or presence of angiotensin (Ang)II (1 μM)**.** NO release was estimated from the area under the curve (AUC) of the fluorescent signal of 4,5-diaminofluorescein (DAF-2) for 30 min of stimulation. Results are shown as mean ± SEM (6-8). **P<0.01 *vs* ctrl; #P<0.05 and ##P<0.01 *vs* AngII.

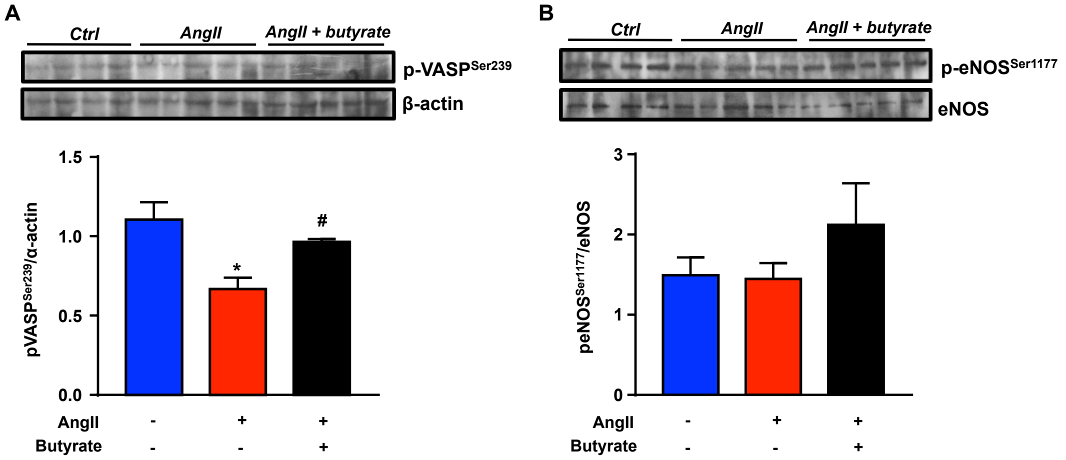

Figure S2. Effects of butyrate in the level of VASP and eNOS phosphorylation in RAECs incubated with angiotensin (Ang)II. Cells were incubated with butyrate (5 mM) for 24 hours, and in the last 6 hours in the absence or presence of angiotensin (Ang)II (1 μM)**.** Results are shown as mean ± SEM (4-5). *P<0.01 *vs* ctrl; #P<0.05 *vs* AngII.

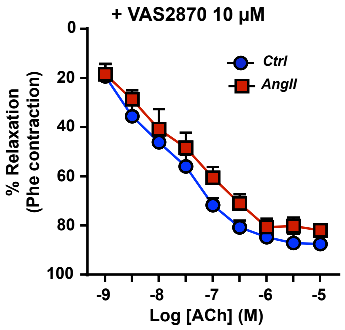

Figure S3. Effects of NADPH oxidase inhibition in angiotensin (Ang)II-induced endothelial dysfunction *in vitro*. Vascular relaxant responses induced by acetylcholine (ACh) in rat aortas pre-contracted by phenylephrine (Phe, 1 µM) incubated with VAS2870 (10 µM), and in the absence (Ctrl) or presence of AngII (1 µM) for 6 hours. Results are shown as mean ± SEM, derived from 6-8 different rings.
